# Supplementary material for: Adaptation to mutational inactivation of an essential gene converges to an accessible suboptimal fitness peak
Source: eLife. 2019 Oct 1;8:e50509. doi: 10.7554/eLife.50509 (PMC6828540; doi:10.7554/eLife.50509)
Supplement: Supplementary file 5. [file elife-50509-supp5.docx]

**Table S1 – Single point mutations in thyA and deoB are predicted to impair catalytic activity and/or destabilize the protein**

| **gene** | **protein** | **mutation** | **predicted ΔΔG^a^** | **Inferred/known effect on catalytic activity or protein stability** | **References** |  |
| --- | --- | --- | --- | --- | --- | --- |
| thyA | thymidylate synthase | L44Q | -1.76 (Reduced stability) | Causes thymine-requiring (Thy-) phenotype of S. flexneri Y strain TSF21 | Nur EKMS, al Mamun AA, & Ahmed ZU (1994) Microbiol Immunol 38(4):309-312. |  |
| thyA | thymidylate synthase | D169G | 0.61 (*Increased stability*) | Active site | Birdsall DL, Finer-Moore J, & Stroud RM (2003) Protein Eng 16(3):229-240. |  |
| thyA | thymidylate synthase | R166C | -0.14 (*Reduced stability*) | Active site | Islam Z, Strutzenberg TS, Ghosh AK, & Kohen A (2015) Acs Catal 5(10):6061-6068. |  |
| thyA | thymidylate synthase | T202P | -1.92 (*Reduced stability*) | Dimer interface | Rengarajan J, et al. (2004) Mol Microbiol 53(1):275-282. |  |
| deoB | phosphopentomutase | D10G | -0.86 (Reduced stability) | Active site residue (D13 in Bacillus cereus DeoB) | Panosian TD, et al. (2011) Journal of Biological Chemistry 286(10):8043-8054. |  |
|  |  |  |  | |  | |

^a^ predicted by <http://marid.bioc.cam.ac.uk/sdm2/run_prediction>

Worth CL, Preissner R, & Blundell TL (2011) SDM-a server for predicting effects of mutations on protein stability and malfunction. Nucleic acids research 39:W215-W222.
