# Supplementary material for: Adaptation to mutational inactivation of an essential gene converges to an accessible suboptimal fitness peak
Source: eLife. 2019 Oct 1;8:e50509. doi: 10.7554/eLife.50509 (PMC6828540; doi:10.7554/eLife.50509)
Supplement: Supplementary file 6. [file elife-50509-supp6.docx]

| **Key Resources Table** | | | | |
| --- | --- | --- | --- | --- |
| **Reagent type (species) or resource** | **Designation** | **Source or reference** | **Identifiers** | **Additional information** |
| Strain, strain background (Escherichia coli) | E coli Strain BW25113/pKD46 | E. coli Genetic Stock Center | strain#: 7739 |  |
| Genetic reagent (Escherichia coli) | *E coli* Strain BW25113 Δ*folA*::(*cmR*-*folA*-*kanR*) | This work |  | See Materials and Methods section Construction of D27 mutants |
| Genetic reagent (Escherichia coli) | *E coli* Strain BW25113 Δ*folA*::(*cmR*-*folA.*p.D27F-*kanR*) | This work |  | See Materials and Methods section Construction of D27 mutants |
| Genetic reagent (Escherichia coli) | *E coli* Strain BW25113 Δ*folA*::(*cmR*-*folA.*p.D27N-*kanR*) | This work |  | See Materials and Methods section Construction of D27 mutants |
| Genetic reagent (Escherichia coli) | *E coli* Strain BW25113 Δ*folA*::(*cmR*-*folA.*p.D27G-*kanR*) | This work |  | See Materials and Methods section Construction of D27 mutants |
| Genetic reagent (Escherichia coli) | *E coli* Strain BW25113 Δ*folA*::(*cmR*-*folA.*p.D27C-*kanR*) | This work |  | See Materials and Methods section Construction of D27 mutants |
| Genetic reagent (Escherichia coli) | TransforMax™ EC100D™ pir+ | Lucigen | Cat#ECP09500 |  |
| Genetic reagent (Escherichia coli) | One Shot™ BL21 Star™ (DE3) Chemically Competent E. coli | Thermofisher | Cat#C601003 |  |
| Chemical compound, drug | Thymine | Sigma | Cat#T0376-10G |  |
| Chemical compound, drug | Inosine | MP biomedicals | Cat#102049 |  |
| Chemical compound, drug | Adenine | Sigma | Cat#A8626 |  |
| Chemical compound, drug | Methionine | Sigma | Cat#64313-25G-F |  |
| Chemical compound, drug | Glycine | Amresco | Cat#0167-1KG |  |
| Chemical compound, drug | Dihydrofolate | Sigma | Cat# D7006-25MG |  |
| Chemical compound, drug | NADPH | RPI | Cat# N20140-0.1 |  |
| Chemical compound, drug | Glucose | Fluka | Cat#49159-1KG |  |
| Chemical compound, drug | Difco M9 minimal salts, 5x | BD | Cat#248510 |  |
| Chemical compound, drug | Chloramphenicol | Calbiochem | Cat#220551 |  |
| Chemical compound, drug | Kanamycin sulphate | Teknova | Cat#K2105 |  |
| Peptide, recombinant protein | Recombinant protein E coli DHFR D27F | This work |  | See Materials and Methods section Protein purification and characterization |
| Peptide, recombinant protein | Recombinant protein E coli DHFR D27N | This work |  | See Materials and Methods section Protein purification and characterization |
| Peptide, recombinant protein | Recombinant protein E coli DHFR D27C | This work |  | See Materials and Methods section Protein purification and characterization |
| Peptide, recombinant protein | Recombinant protein E coli DHFR D27G | This work |  | See Materials and Methods section Protein purification and characterization |
| Peptide, recombinant protein | KOD Hot Start DNA Polymerase | Sigma-Aldrich | Cat#71086 |  |
| Peptide, recombinant protein | Phusion High-Fidelity DNA Polymerase | Thermofisher scientific | Cat#F530S |  |
| Commercial assay, kit | DNA Clean & Concentrator (DCC) | Zymo research | Cat#D4013 |  |
| Recombinant DNA reagent | Plasmid pKD13 | E. coli Genetic Stock Center (9) | GenBank: AY048744.1 |  |
| Recombinant DNA reagent | Plasmid pKD13-cmR-folA(wt) | (Bershtein et al., 2012) |  |  |
| Recombinant DNA reagent | Plasmid pKD13-*kefC*(897-1863)-*cmR*-folA(wt)-*kanR-apaH-apaG* | This work |  | See Materials and Methods section Construction of Plasmid pKD13-kefC(897-1863)-cmR-folA(wt)-kanR-apaH-apaG |
| Recombinant DNA reagent | Plasmid pKD13-*kefC*(897-1863)-*cmR*-folA(p.D27F)-*kanR-apaH-apaG* | This work |  | See Materials and Methods section Mutations in D27 folA locus |
| Recombinant DNA reagent | Plasmid pKD13-*kefC*(897-1863)-*cmR*-folA(p.D27N)-*kanR-apaH-apaG* | This work |  | See Materials and Methods section Mutations in D27 folA locus |
| Recombinant DNA reagent | Plasmid pKD13-*kefC*(897-1863)-*cmR*-folA(p.D27G)-*kanR-apaH-apaG* | This work |  | See Materials and Methods section Mutations in D27 folA locus |
| Recombinant DNA reagent | Plasmid pKD13-*kefC*(897-1863)-*cmR*-folA(p.D27C)-*kanR-apaH-apaG* | This work |  | See Materials and Methods section Mutations in D27 folA locus |
| Recombinant DNA reagent | Plasmid pEM-Cas9HF1-recA56 | Addgene | Addgene plasmid # 89962 |  |
| Recombinant DNA reagent | Plasmid pTRC-thyA | (Bhattacharyya et al., 2016) |  |  |
| Recombinant DNA reagent | Plasmid pTRC-*tetR*-deoB(wt) | This work |  | See Materials and Methods section  Effect of DeoB and ThyA expression |
| Recombinant DNA reagent | Plasmid pTRC-*tetR*-deoB(p.309STOP) | This work |  | See Materials and Methods section  Effect of DeoB and ThyA expression |
| Recombinant DNA reagent | Plasmid pTRC-*tetR*-*thyA*(wt) | This work |  | See Materials and Methods section  Effect of DeoB and ThyA expression |
| Recombinant DNA reagent | Plasmid pTRC-*tetR*-thyA(755-762del) | This work |  | See Materials and Methods section  Effect of DeoB and ThyA expression |
| Software, algorithm | Proteomics Grouping analysis, Matlab Code | Bershtein, Choi, et al., 2015 |  |  |
| Software, algorithm | Evolution Script, Freedom EVOware software | This work |  |  |
| Software, algorithm | Matlab R2018a | Mathworks | https://www.mathworks.com/products/matlab.html |  |
| Software, algorithm | Origin Pro7 | OriginLab | https://www.originlab.com/ |  |
| Software, algorithm | Freedom EVOware | Tecan | https://lifesciences.tecan.com/software-freedom-evoware |  |
| Software, algorithm | Evolution Script, Freedom EVOware software | This work |  |  |
| Software, algorithm | MzMatch | (Scheltema, Jankevics, Jansen, Swertz, & Breitling, 2011) | http://mzmatch.sourceforge.net/installation.php |  |
| Software, algorithm | R 3.5.0 | The R Project for Statistical Computing | https://www.r-project.org/ |  |
| Software, algorithm | Ideom V19 | (Creek, Jankevics, Burgess, Breitling, & Barrett, 2012) | http://mzmatch.sourceforge.net/ideom.php |  |
| Other | Freedom Evo 75 | Tecan | https://lifesciences.tecan.com/products/liquid_handling_and_automation/freedom_evo_series |  |
| Other | Infinite® 200 PRO | Tecan | https://lifesciences.tecan.com/plate_readers/infinite_200_pro |  |
| Other | Liconic STX44 | Liconic | https://www.liconic.com/index.php |  |
| Sequence-based reagent | tetR_pTRC_for | Sigma-Aldrich |  | ACACCATCGAATGATATCGACGTCTTAAGACCCACTTT |
| Sequence-based reagent | tetR_thyA_rev | Sigma-Aldrich |  | CTAAATACTGTTTCATAGATCCGAAGTCCTCTTTAGATC |
| Sequence-based reagent | tetR_thyA_for | Sigma-Aldrich |  | GACTTCGGATCTATGAAACAGTATTTAGAACTGATGC |
| Sequence-based reagent | tetR_thyA-rev | Sigma-Aldrich |  | GACTTCGGATCTATGAAACAGTATTTAGAACTGATGC |
| Sequence-based reagent | tetR_deoB_for | Sigma-Aldrich |  | GACTTCGGATCTATGAAACGTGCATTTATTATGGTGC |
| Sequence-based reagent | pTRC_deoB_rev | Sigma-Aldrich |  | AAAACAGCCAAGCTTTCAGAACATGGCTTTGCCATATTCC |
| Sequence-based reagent | deoB_pTRC_for | Sigma-Aldrich |  | AAAGCCATGTTCTGAAAGCTTGGCTGTTTTGGCGGATGAG |
| Sequence-based reagent | deoB_tetR_rev | Sigma-Aldrich |  | AAATGCACGTTTCATAGATCCGAAGTCCTCTTTAGATC |
| Sequence-based reagent | CapR-Chrom-Flanking rev | Sigma-Aldrich |  | TTAGGATGAGGGTTTCGTTTCCGGTTCATC |
| Sequence-based reagent | PCRseq_KefC_for2 | Sigma-Aldrich |  | CTGCTCGGTTTCCTCATCATCAA |
| Sequence-based reagent | P4_chrom_flanking for | Sigma-Aldrich |  | TTTTGTATAGAATTTACGGCTAGCGCCG |
| Sequence-based reagent | PCRseq_apaH_rev | Sigma-Aldrich |  | CGTCCCTTTCAGCATCGACATT |
| Sequence-based reagent | pKD13_post_downsream_for | Sigma-Aldrich |  | CTTATCACTGATCAGTGAATTAATGGCG |
| Sequence-based reagent | pKD13_post_upstream_rev | Sigma-Aldrich |  | GACAATAACCCTGATAAATGCTTCAATAATATTG |
| Sequence-based reagent | Upstream_capR_for | Sigma-Aldrich |  | GAAGAAGGTAAACATACCGGCAACATGGCGGATGAACCGGAAACGAAACCCTCATCCTAATCATGATCATCGCAGTACTGTTG |
| Sequence-based reagent | P4downstream_rev | Sigma-Aldrich |  | AAGGCCGGATAAGACGCGACCGGCGTCGCATCCGGCGCTAGCCGTAAATTCTATACAAAACTGTCAAACATGAGAATTAATTC |
| Sequence-based reagent | Ampl_RRfolA_for | Sigma-Aldrich |  | GTGCCGATCAACGTCTCATTTTCG |
| Sequence-based reagent | Ampl_RRfolA_rev | Sigma-Aldrich |  | GCTTCCTCGTGCTTTACGGTATCG |
| Sequence-based reagent | PCRseq_RRfolA_rev | Sigma-Aldrich |  | GCCTTCTATCGCCTTCTTGACGA |
| Sequence-based reagent | D27Fmut_For | Sigma-Aldrich |  | TTTCTCGCCTGGTTTAAACGCAACACCTTAAATAAAC |
| Sequence-based reagent | D27Gmut_for | Sigma-Aldrich |  | GGCCTCGCCTGGTTTAAACGCAACACCTTAAATAAA |
| Sequence-based reagent | D27Nmut_for | Sigma-Aldrich |  | AATCTCGCCTGGTTTAAACGCAACACCTTAAATAAAC |
| Sequence-based reagent | D27_rev | Sigma-Aldrich |  | GGCAGGCAGGTTCCACGGCATGG |
