# Supplementary material for: Adaptation to mutational inactivation of an essential gene converges to an accessible suboptimal fitness peak
Source: eLife. 2019 Oct 1;8:e50509. doi: 10.7554/eLife.50509 (PMC6828540; doi:10.7554/eLife.50509)
Supplement: Source code 1. [file elife-50509-code1.zip › Evolution script.zip!/AppendLOG.docx]

```
'AppendLog.vbs

dim fs, CurrentPhaseFile
Dim objFolder, objFile, dilfile Dim i
Dim DoPause, dilutions, passage,	plate, column, stockstr,	stockdilstr, last_dilutiontime, last_dilutiontimeNumber,last_dilutionOD_Str
Dim CurrODStr, currRate, setOD1,	reference_rate, timestr, timenumber, VolCellsStr,sumRatesStr, rateCountStr
Const ForReading = 1, ForWriting = 2, ForAppending = 8

samplenum = wScript.Arguments(0)

Set fs = CreateObject("Scripting.FileSystemObject")


- Read dilution file
set dilFile= fs.OpenTextFile("C:\Joao\Results\Evolution_data\dilution" & samplenum &
".csv", ForReading,1)
DoPause = CInt(dilFile.ReadLine) dilutions = CInt(dilFile.ReadLine) passage = CInt(dilFile.ReadLine) plate = CInt(dilFile.ReadLine) column = CInt(dilFile.ReadLine) stockstr = dilFile.ReadLine stockdilstr = dilFile.ReadLine
last_dilutionOD_Str = dilFile.ReadLine last_dilutiontimeNumber = dilFile.ReadLine CurrODStr = dilFile.ReadLine
currRate = dilFile.ReadLine setOD1 = dilFile.ReadLine reference_rate = dilFile.ReadLine timestr = dilFile.ReadLine
timenumber = Cdbl(dilFile.ReadLine) VolCellsStr = dilFile.ReadLine rateCountStr = dilFile.ReadLine sumRatesStr =  dilFile.ReadLine avOD = dilFile.ReadLine
dilFile.close

Set CurrentPhaseFile= fs.CreateTextFile("C:\Joao\Results\Evolution_data\current_phase" &
samplenum & ".txt")
If DoPause = 1 then 'write current phase file

CurrentPhaseFile.writeline 0 CurrentPhaseFile.writeline passage CurrentPhaseFile.writeline plate CurrentPhaseFile.writeline column CurrentPhaseFile.writeline stockstr CurrentPhaseFile.writeline stockdilstr CurrentPhaseFile.writeline last_dilutionOD_Str CurrentPhaseFile.writeline last_dilutiontimeNumber CurrentPhaseFile.writeline setOD1 CurrentPhaseFile.writeline reference_rate CurrentPhaseFile.WriteLine rateCountStr CurrentPhaseFile.WriteLine sumRatesStr CurrentPhaseFile.WriteLine avOD CurrentPhaseFile.close

else
CurrentPhaseFile.writeline DoPause CurrentPhaseFile.writeline passage CurrentPhaseFile.writeline plate


end If
```

CurrentPhaseFile.writeline column CurrentPhaseFile.writeline stockstr CurrentPhaseFile.writeline stockdilstr CurrentPhaseFile.writeline last_dilutionOD_Str CurrentPhaseFile.writeline last_dilutiontimeNumber CurrentPhaseFile.writeline setOD1 CurrentPhaseFile.writeline reference_rate CurrentPhaseFile.WriteLine rateCountStr CurrentPhaseFile.WriteLine sumRatesStr CurrentPhaseFile.WriteLine avOD CurrentPhaseFile.close

set Logfile**=** fs.OpenTextFile**(**"C:\Joao\Results\Evolution_data\Logfile" **&** samplenum **&** ".csv"**,** Forappending**)**

Logfile.writeline timestr **&** "," **&** timenumber **&** "," **&** dilutions **&** "," **&** passage **&** "," **&** plate **&** "," **&** column **&** "," **&** stockstr **&** "," **&** stockdilstr **&** "," **&** last_dilutionOD_Str **&** "," **&** last_dilutiontimeNumber **&** "," **&** CurrODStr **&** "," **&** currRate **&** "," **&** setOD1 **&** "," **&** reference_rate **&** "," **&** VolCellsStr& "," **&** avOD

LogFile.close

set LogFile **=** Nothing

set fs **=** Nothing
