# Supplementary material for: Adaptation to mutational inactivation of an essential gene converges to an accessible suboptimal fitness peak
Source: eLife. 2019 Oct 1;8:e50509. doi: 10.7554/eLife.50509 (PMC6828540; doi:10.7554/eLife.50509)
Supplement: Source code 1. [file elife-50509-code1.zip › Evolution script.zip!/AppendLOG_dilutions.docx]

```
'AppendLOG_dilutions  dim fs, CurrentPhaseFile
Dim objFolder, objFile, dilfile,ODfile Dim i
Dim DoPause, dilutions, passage,	plate, column, stockstr, stockdilstr, last_dilutiontime, last_dilutionOD_Str, currODDbl(8),ODStr,arrStr,tempStr,datestr
Dim CurrODStr, currRate, setOD1, reference_rate, timestr, timenumber,arrOD(8),temp_t, VolCellsStr,currRatedil
Const ForReading = 1, ForWriting = 2, ForAppending = 8

filename = wScript.Arguments(0) samplenum = wScript.Arguments(1)


Set fs = CreateObject("Scripting.FileSystemObject")


'10 - Read dilution file
set dilFile= fs.OpenTextFile("C:\Joao\Results\Evolution_data\dilution" & samplenum &
".csv", ForReading,1)
DoPause = CInt(dilFile.ReadLine) dilutions = CInt(dilFile.ReadLine) passage = CInt(dilFile.ReadLine)

plate = CInt(dilFile.ReadLine) column = CInt(dilFile.ReadLine) stockstr = dilFile.ReadLine stockdilstr = dilFile.ReadLine dilFile.ReadLine dilFile.ReadLine dilFile.ReadLine
currRate = dilFile.ReadLine setOD1 = dilFile.ReadLine

reference_rate = dilFile.ReadLine dilFile.ReadLine  dilFile.ReadLine
VolCellsStr = dilFile.ReadLine rateCountStr = dilFile.ReadLine sumRatesStr = dilFile.ReadLine avOD = dilFile.ReadLine
dilFile.close


column = column +1 passage = passage + 1 currRatedil = currRate if column > 12 then
column = 1
plate = plate + 1
end if


'Read OD file

set ODfile= fs.OpenTextFile("C:\Joao\Results\Evolution_data\asc\" & filename & ".asc", ForReading)

ODStr = ""
arrStr= "" tempStr =""
arrStr = ODfile.ReadLine
tempStr = split(ODfile.ReadLine,"measurement: ") datestr = split(tempStr(1),"/")
timestr = tempStr(2)
timenumber = (Cdbl(cdate(datestr(0)))+cdbl(cdate(timestr)))*24
timestr = datestr(0) & " " & tempStr(2) ODStr = split(arrStr,",")

CurrODStr = "" avOD = 0

for i = 1 to 8
arrOD(i) = (CDbl(ODStr(i+8*(column-1)))-0.082)*2.22
CurrODStr = CurrODStr & Cstr(arrOD(i)) & "," avOD = avOD+arrOD(i)
next
CurrODStr = Left(CurrODStr, Len(CurrODStr)-1) avOD = 0
for i = 2 to 7
avOD = avOD+arrOD(i)
next
avOD = avOD/6 ODfile.Close
set ODfile = Nothing

last_dilutionOD_Str = CurrODStr last_dilutiontimeNumber = timenumber


'write current phase file
set CurrentPhaseFile= fs.CreateTextFile("C:\Joao\Results\Evolution_data\current_phase" &
samplenum &  ".txt") CurrentPhaseFile.writeline 0 CurrentPhaseFile.writeline passage CurrentPhaseFile.writeline plate CurrentPhaseFile.writeline column CurrentPhaseFile.writeline stockstr CurrentPhaseFile.writeline stockdilstr CurrentPhaseFile.writeline last_dilutionOD_Str
CurrentPhaseFile.writeline last_dilutiontimeNumber CurrentPhaseFile.writeline setOD1 CurrentPhaseFile.writeline reference_rate CurrentPhaseFile.writeline "0,0,0,0,0,0,0,0"
CurrentPhaseFile.writeline "0,0,0,0,0,0,0,0" CurrentPhaseFile.WriteLine avOD
CurrentPhaseFile.close


Forappending)
Logfile.writeline timestr & "," & timenumber & "," & dilutions & "," & passage & "," & plate & "," & column & "," & stockstr & "," & stockdilstr & "," & last_dilutionOD_Str & "," & last_dilutiontimeNumber & "," & CurrODStr & "," & currRate & "," & setOD1 & ","  & reference_rate & "," & VolCellsStr	& "," & currRatedil

LogFile.close
set LogFile = Nothing


set dilFile = fs.CreateTextFile("C:\Joao\Results\Evolution_data\dilution" & samplenum & ".csv") dilFile.writeline 0
dilFile.writeline  0 dilFile.writeline passage dilFile.writeline plate dilFile.writeline column dilFile.writeline stockstr dilFile.writeline stockdilstr dilFile.writeline last_dilutionOD_Str
dilFile.writeline last_dilutiontimeNumber dilFile.writeline CurrODStr dilFile.writeline currRate dilFile.writeline setOD1 dilFile.writeline reference_rate dilFile.writeline timestr dilFile.writeline timenumber dilFile.writeline "0,0,0,0,0,0,0,0"
dilFile.writeline "0,0,0,0,0,0,0,0"
dilFile.writeline "0,0,0,0,0,0,0,0" dilFile.writeline avOD


dilFile.close set fs = Nothing
```
