# Supplementary material for: Adaptation to mutational inactivation of an essential gene converges to an accessible suboptimal fitness peak
Source: eLife. 2019 Oct 1;8:e50509. doi: 10.7554/eLife.50509 (PMC6828540; doi:10.7554/eLife.50509)
Supplement: Source code 1. [file elife-50509-code1.zip › Evolution script.zip!/calculate_dilutions.docx]

```
'Calculate_dilutions.vbs

dim fs, objtextfile,CurrentPhaseFile, ODfile
Dim objFolder, objFile, dilfile, strNewName, strOldName Dim strPath, strName, i
dim ODStr, avOD, arrStr, tempstr, dilution
dim DoPause, passage,	plate, column, stockstr, stockdilstr, last_dilutionOD(8),last_dilutiontimeNumber, last_dilutionOD_Str, rateCount(8), rateCountStr dim CurrODStr, currRate(8),setOD1,referrence_rate,timestr,timenumber, currentAntibioticStock(8),currentAntibioticDil(8), VolCells(8), VolCellsStr, ODboolean
dim arrOD(8),sumRates(8), sumRatesStr, avRate(8) dim newfs, outputname


filename = wScript.Arguments(0) samplenum = wScript.Arguments(1)


Const ForReading = 1, ForWriting = 2, ForAppending = 8


Set fs = CreateObject("Scripting.FileSystemObject") 'Read current_phase file
'1	DoPause	Pause experiment? 0= no	1=yes
'2	passage	Passage number
'3	plate	plate number
'4	column	Column of 96-well plate in use (1-12)
'5	stockstr	Stock solutions of folAmix. The position and concentations are the following 1= 1x, 2 = 0.125x, 3=0.015625x, 4=0.001953125,5=0.000244141
'6	stockdilstr	Volume of folAmix to pipete (2.5,5,10 uL)
'7	last_dilutionOD_Str	array (1-8) with the ODs of each well after the last dilution '8	last_dilutiontimeNumber time of last dilution
'9	setOD1	OD threshold for dilutions '10	referrence_rate	Reference growth rate
'11	rateCount	array(1-8) with sum of OD values when OD was between 0.1 and 0.25 '12	sumRatesStr	array(1-8) with number of measurements that OD was between 0.1 and 0.25
'13	avOD	average OD of all wells

set CurrentPhaseFile= fs.OpenTextFile("C:\Joao\Results\Evolution_data\current_phase" &
samplenum & ".txt", ForReading,1)
DoPause = CInt(CurrentPhaseFile.ReadLine) passage = CInt(CurrentPhaseFile.ReadLine) plate = CInt(CurrentPhaseFile.ReadLine) column = CInt(CurrentPhaseFile.ReadLine) stockstr = CurrentPhaseFile.ReadLine stockdilstr = CurrentPhaseFile.ReadLine
last_dilutionOD_Str = CurrentPhaseFile.ReadLine last_dilutiontimeNumber = Cdbl(CurrentPhaseFile.ReadLine) setOD1 = Cdbl(CurrentPhaseFile.ReadLine)
referrence_rate = Cdbl(CurrentPhaseFile.ReadLine) rateCountStr = CurrentPhaseFile.ReadLine sumRatesStr = CurrentPhaseFile.ReadLine CurrentPhaseFile.close
set CurrentPhaseFile = Nothing

tempstr= ""
tempstr = split(stockstr, ",") for i = 1 to 8
currentAntibioticStock(i) = CInt(tempstr(i-1)) next

tempstr= ""
tempstr = split(stockdilstr, ",") for i = 1 to 8
currentAntibioticDil(i) = Cdbl(tempstr(i-1)) next
tempstr= ""
tempstr = split(last_dilutionOD_Str, ",") for i = 1 to 8
last_dilutionOD(i) = Cdbl(tempstr(i-1))
Next

tempstr= ""
tempstr = split(rateCountStr, ",") for i = 1 to 8
rateCount(i) = CInt(tempstr(i-1))
Next tempstr= ""
tempstr = split(sumRatesStr, ",") for i = 1 to 8
sumRates(i) = CDbl(tempstr(i-1))
Next

'Read OD file

set ODfile= fs.OpenTextFile("C:\Joao\Results\Evolution_data\asc\" & filename & ".asc", ForReading)
ODStr = ""
arrStr= "" tempStr =""
arrStr = ODfile.ReadLine
tempStr = split(ODfile.ReadLine,"measurement: ") datestr = split(tempStr(1),"/")
timestr = tempStr(2)
timenumber = (Cdbl(cdate(datestr(0)))+cdbl(cdate(timestr)))*24
timestr = datestr(0) & " " & tempStr(2) ODStr = split(arrStr,",")
CurrODStr = "" avOD = 0
ODboolean = 1
for i = 1 to 8
arrOD(i) = (CDbl(ODStr(i+8*(column-1)))-0.082)*2.22	'subtract blank and correct optical density to 1cm pathlength
CurrODStr = CurrODStr & Cstr(arrOD(i)) & "," next
CurrODStr = Left(CurrODStr, Len(CurrODStr)-1) ' select wells for calculations
avOD = 0
for i = 2 to 7
avOD = avOD+arrOD(i)
Next
avOD = avOD/6 ODfile.Close
set ODfile = Nothing
'Compare average OD with threshold if avOD > setOD1 then
dilution =1
else
if (timenumber-last_dilutiontimeNumber)>20 then dilution = 1
else
dilution =0
end if

end if


'dilutions flile structure
'1	DoPause	Pause experiment? 0= no	1=yes
'2	dilution	Perform dilutions ? 0= no	1=yes
'3	passage	Passage number
'4	plate	plate number
'5	column	Column of 96-well plate in use (1-12)
'6	stockstr	Stock solutions of folAmix. The position and concentations are the following 1= 1x, 2 = 0.125x, 3=0.015625x, 4=0.001953125,5=0.000244141
'7	stockdilstr	Volume of folAmix to pipete (2.5,5,10 uL)
'8	last_dilutionOD_Str	array (1-8) with the ODs of each well after the last dilution '9	last_dilutiontimeNumber time of last dilution
'10	CurrODStr	array (1-8) with current ODs
'11	currRateStr	array (1-8) with current growth rate '12	setOD1	OD threshold for dilutions
'13	referrence_rate	Reference growth rate '14	timestr	Current date/time
'15	timenumber	Current date/time in number format
'16	VolCellsStr	array (1-8) with volume of cells to pipette
'17	rateCountStr	array(1-8) with sum of OD values when OD was between 0.1 and 0.25 '18	sumRatesStr	array(1-8) with number of measurements that OD was between 0.1 and 0.25
'19	avOD	average OD of all wells


set dilFile = fs.CreateTextFile("C:\Joao\Results\Evolution_data\dilution" & samplenum & ".csv") stockstr = ""
stockdilstr ="" currRateStr = "" VolCellsStr = "" sumRatesStr = "" rateCountStr =  "" if dilution = 0 then
if passage = 0 then for i = 1 to 8
currRate(i) = 0
currRateStr = currRateStr & currRate(i) & ","
stockdilstr = stockdilstr & Cstr(currentAntibioticDil(i)) & "," stockstr = stockstr & Cstr(currentAntibioticStock(i)) & "," sumRatesStr = sumRatesStr & CStr(sumRates(i)) & "," rateCountStr = rateCountStr & CStr(rateCount(i)) & ","
next
currRateStr = Left(currRateStr,Len(currRateStr)-1) stockdilstr = Left(stockdilstr,Len(stockdilstr)-1) stockstr =  Left(stockstr,Len(stockstr)-1) sumRatesStr = Left(sumRatesStr,Len(sumRatesStr)-1) rateCountStr = Left(rateCountStr,Len(rateCountStr)-1)
else
for i = 1 to 8
If last_dilutionOD(i) < 0.001 Then last_dilutionOD(i) = 0.001
End If
If arrOD(i) < 0.001 Then arrOD(i) = 0.001
End If currRate(i) =
log(arrOD(i)/last_dilutionOD(i))/(timenumber-last_dilutiontimeNumber)

stockdilstr = stockdilstr & Cstr(currentAntibioticDil(i)) & "," stockstr = stockstr & Cstr(currentAntibioticStock(i)) & ","
If (arrOD(i)> 0.14 And arrOD(i)< 0.225) Then

sumRates(i) = sumRates(i) + currRate(i) rateCount(i) = rateCount(i)+1
sumRatesStr = sumRatesStr & CStr(sumRates(i)) & "," rateCountStr = rateCountStr & CStr(rateCount(i)) & ","
Else
sumRatesStr = sumRatesStr & CStr(sumRates(i)) & "," rateCountStr = rateCountStr & CStr(rateCount(i)) & ","
End If

If rateCount(i) = 0 Then avRate(i) = currRate(i)
Else
avRate(i) = sumRates(i)/rateCount(i)
End If
currRateStr = currRateStr & CStr(avRate(i)) & "," next
currRateStr = Left(currRateStr,Len(currRateStr)-1) stockdilstr = Left(stockdilstr,Len(stockdilstr)-1) stockstr =  Left(stockstr,Len(stockstr)-1) sumRatesStr = Left(sumRatesStr,Len(sumRatesStr)-1) rateCountStr = Left(rateCountStr,Len(rateCountStr)-1)
end if
'update dilution file dilFile.writeline DoPause dilFile.writeline dilution dilFile.writeline passage dilFile.writeline plate dilFile.writeline column dilFile.writeline stockstr dilFile.writeline stockdilstr dilFile.writeline last_dilutionOD_Str
dilFile.writeline last_dilutiontimeNumber dilFile.writeline CurrODStr dilFile.writeline currRateStr dilFile.writeline setOD1 dilFile.writeline referrence_rate dilFile.writeline timestr dilFile.writeline timenumber dilFile.writeline "0,0,0,0,0,0,0,0" dilFile.WriteLine rateCountStr dilFile.WriteLine sumRatesStr dilFile.WriteLine avOD
end if


if dilution = 1 then ' calculate rate
if passage < 2 then for i = 1 to 8
If last_dilutionOD(i) < 0.001 Then ' if OD is below detection limit, the OD used for calculations is OD=0.001
last_dilutionOD(i) = 0.001
End If
If arrOD(i) < 0.001 Then arrOD(i) = 0.001
End If currRate(i) =
log(arrOD(i)/last_dilutionOD(i))/(timenumber-last_dilutiontimeNumber) 'Calculate growth rate
stockdilstr = stockdilstr & Cstr(currentAntibioticDil(i)) & "," stockstr = stockstr & Cstr(currentAntibioticStock(i)) & ","
If (arrOD(i)> 0.1 And arrOD(i)< 0.25) Then ' OD measurement are considered for growth rate calculations if within a defined range
sumRates(i) = sumRates(i) + currRate(i)

rateCount(i) = rateCount(i)+1
sumRatesStr = sumRatesStr & CStr(sumRates(i)) & "," rateCountStr = rateCountStr & CStr(rateCount(i)) & ","
Else
sumRatesStr = sumRatesStr & CStr(sumRates(i)) & "," rateCountStr = rateCountStr & CStr(rateCount(i)) & ","
End If
If rateCount(i) = 0 Then avRate(i) = currRate(i)
Else
avRate(i) = sumRates(i)/rateCount(i)
End If
currRateStr = currRateStr & CStr(avRate(i)) & ","
VolCells(i) = round((2)/arrOD(i)) 'Calculate volume of cells to pipet to next passage for a starting OD of 0.01.
If VolCells(i) >50 Then
VolCells(i) =50 'maximum volume of cells set to 50 uL End if
If VolCells(i) <5 Then
VolCells(i) =5 ' minimum volume of cells set to 5 uL end If

' Volume of cells in 1st and 8th wells
VolCellsStr = VolCellsStr & Cstr(VolCells(i)) & "," next
currRateStr = Left(currRateStr,Len(currRateStr)-1) stockdilstr = Left(stockdilstr,Len(stockdilstr)-1) stockstr =  Left(stockstr,Len(stockstr)-1) VolCellsStr	= Left(VolCellsStr, Len(VolCellsStr)-1) sumRatesStr = Left(sumRatesStr,Len(sumRatesStr)-1) rateCountStr = Left(rateCountStr,Len(rateCountStr)-1)
end if
if passage > 1 then
if passage = 2 then referrence_rate = 0
for i = 1 to 8
If last_dilutionOD(i) < 0.001 Then last_dilutionOD(i) = 0.001
End If
If arrOD(i) < 0.001 Then arrOD(i) = 0.001
End If currRate(i) =
log(arrOD(i)/last_dilutionOD(i))/(timenumber-last_dilutiontimeNumber)

If (arrOD(i)> 0.1 And arrOD(i)< 0.25) Then sumRates(i) = sumRates(i) + currRate(i) rateCount(i) = rateCount(i)+1
sumRatesStr = sumRatesStr & CStr(sumRates(i)) & "," rateCountStr = rateCountStr & CStr(rateCount(i)) & ","
Else
sumRatesStr = sumRatesStr & CStr(sumRates(i)) & "," rateCountStr = rateCountStr & CStr(rateCount(i)) & ","
End If
If rateCount(i) = 0 Then avRate(i) = currRate(i)
Else
avRate(i) = sumRates(i)/rateCount(i)
End If
currRateStr = currRateStr & CStr(avRate(i)) & "," ' set reference rate
referrence_rate = referrence_rate + avRate(i) ' Start decreasing folAmix
currentAntibioticStock(i) = currentAntibioticStock(i)

stockdilstr = stockdilstr & Cstr(currentAntibioticDil(i)) & "," stockstr = stockstr & Cstr(currentAntibioticStock(i)) & ","
' calculate volume of cells VolCells(i) = round((2)/arrOD(i)) if VolCells(i) >50 Then
VolCells(i) =50
End if
If VolCells(i) <5 Then VolCells(i) =5
End If
' Volume of cells in 1st and 8th wells VolCellsStr = VolCellsStr & Cstr(VolCells(i)) & ","
next
referrence_rate = referrence_rate/6
currRateStr = Left(currRateStr,Len(currRateStr)-1) stockdilstr = Left(stockdilstr,Len(stockdilstr)-1) stockstr =  Left(stockstr,Len(stockstr)-1) VolCellsStr	= Left(VolCellsStr, Len(VolCellsStr)-1) sumRatesStr = Left(sumRatesStr,Len(sumRatesStr)-1) rateCountStr = Left(rateCountStr,Len(rateCountStr)-1)
Else
for i = 1 to 8
If last_dilutionOD(i) < 0.001 Then last_dilutionOD(i) = 0.001
End If
If arrOD(i) < 0.001 Then arrOD(i) = 0.001
End If currRate(i) =
log(arrOD(i)/last_dilutionOD(i))/(timenumber-last_dilutiontimeNumber)

If (arrOD(i)> 0.1 And arrOD(i)< 0.25) Then sumRates(i) = sumRates(i) + currRate(i) rateCount(i) = rateCount(i)+1
sumRatesStr = sumRatesStr & CStr(sumRates(i)) & "," rateCountStr = rateCountStr & CStr(rateCount(i)) & ","
Else
sumRatesStr = sumRatesStr & CStr(sumRates(i)) & "," rateCountStr = rateCountStr & CStr(rateCount(i)) & ","
End If
If rateCount(i) = 0 Then avRate(i) = currRate(i)
Else
avRate(i) = sumRates(i)/rateCount(i)
End If
currRateStr = currRateStr & CStr(avRate(i)) & ","
If (avRate(i)/referrence_rate>0.64 and arrOD(i)>0.1) then currentAntibioticDil(i) = currentAntibioticDil(i)/2 if currentAntibioticDil(i)<2.5 then
if currentAntibioticStock(i)<6 then currentAntibioticStock(i) = currentAntibioticStock(i)+1 currentAntibioticDil(i) = 10
else
currentAntibioticStock(i) = 6
currentAntibioticDil(i) = 0
end if end if
else
if (avRate(i)/referrence_rate<0.01) Then if currentAntibioticStock(i) >1 then
currentAntibioticDil(i) = currentAntibioticDil(i)*2 if currentAntibioticDil(i)>10 then
currentAntibioticStock(i) = currentAntibioticStock(i)-1

currentAntibioticDil(i) = 2.5
end if else
currentAntibioticStock(i) = 1
end if else
currentAntibioticStock(i) = currentAntibioticStock(i) end if
end if
currentAntibioticDil(i) = currentAntibioticDil(i)
stockdilstr = stockdilstr & Cstr(currentAntibioticDil(i)) & "," stockstr = stockstr & Cstr(currentAntibioticStock(i)) &
","
' calculate volume of cells VolCells(i) = round((2)/arrOD(i)) if VolCells(i) >50 Then
VolCells(i) =50
End if
If VolCells(i) <5 Then VolCells(i) =5
end If
' Volume of cells in 1st and 8th wells
VolCellsStr = VolCellsStr & Cstr(VolCells(i)) & "," next
currRateStr = Left(currRateStr,Len(currRateStr)-1) stockdilstr = Left(stockdilstr,Len(stockdilstr)-1) stockstr =  Left(stockstr,Len(stockstr)-1) VolCellsStr	= Left(VolCellsStr, Len(VolCellsStr)-1) sumRatesStr = Left(sumRatesStr,Len(sumRatesStr)-1) rateCountStr = Left(rateCountStr,Len(rateCountStr)-1)
end if end if
dilFile.writeline DoPause dilFile.writeline dilution dilFile.writeline passage dilFile.writeline plate dilFile.writeline column dilFile.writeline stockstr dilFile.writeline stockdilstr dilFile.writeline last_dilutionOD_Str
dilFile.writeline last_dilutiontimeNumber dilFile.writeline CurrODStr dilFile.writeline currRateStr dilFile.writeline setOD1 dilFile.writeline referrence_rate dilFile.writeline timestr dilFile.writeline timenumber dilFile.writeline VolCellsStr dilFile.WriteLine rateCountStr dilFile.WriteLine sumRatesStr dilFile.WriteLine avOD
end if dilFile.close set fs = Nothing
```
