# Supplementary material for: Adaptation to mutational inactivation of an essential gene converges to an accessible suboptimal fitness peak
Source: eLife. 2019 Oct 1;8:e50509. doi: 10.7554/eLife.50509 (PMC6828540; doi:10.7554/eLife.50509)
Supplement: Source code 1. [file elife-50509-code1.zip › Evolution script.zip!/Main script.docx]

**General procedure of main script to run the serial transfer evolution protocol**

The main script was implemented in Freedom EVOware (Tecan) software controlling a Freedom Evo 75 (Tecan) liquid handler equipped with an Infinite® 200 PRO (Tecan) plate reader and a Liconic STX44 shaker. The entire procedure is summarized below:

Load Plates on Shaker

Start shaking

Set temperature = 30C

Loop

For sample i= 1 to 4

Get plate i from shaker

ReadOD (measurements saved to ASCII file *.asc)

Execute external script calculate_dilutions.vbs

-input arguments(filename = ODmeasurement_filename.asc, sample = i)

-output file dilution(i).csv

Read dilution file (dilution(i).csv), (variable in line 2 = dilution)

If dilution = 1

Read dilution file (dilution(i).csv)

Import variables (current column, Volume of cells, Volume of folAmix)

Volume of M9 = 200 – Volume of cells – Volume of folAmix

Pipette M9

Pipette FolAmix

Pipette Cells

ReadOD (measurements saved as text file)

Execute external script AppendLOG_dilutions.vbs

-input arguments(filename = ODmeasurement_filename.asc, sample = i)

- updates current_phase(i).txt

-updates logfile(i).csv

end

If dilution = 0

Execute external script appendLOG.vbs

-input arguments(filename = ODmeasurements.txt, sample = i)

- updates currentphase file

-updates logfile

End

Load plate i on Shaker

Wait 30 min

Next sample

The following external scripts were generated in VBscript:

calculate_dilutions.vbs	This script calculates the corrected optical density and the growth rate for every well and compares the average OD of all wells to decide whether a new serial transfer passage should occur (dilution =1) or not (dilution =0). It also makes decision whether to maintain, increase or decrease the concentration of folAmix based on the growth rate for each well.

AppendLOG.vbs	This script updates the logfile of each sample with the most recent data

AppendLOG_dilutions.vbs	The same as AppendLOG.vbs, but runs different instructions after a new serial transfer occurs

The files needed to run the experiment are stored in a single directory. Three different files are needed for each sample running (96-well plate).

e.g. for sample 1:

\current_phase1.txt	‘this file has stored information on the passage number, column currently being used, the position of stock solutions of folAmix currently in use and volume of folAmix to pipette for each well in the column.

\dilution1.csv	‘this file serves as temporary file that the liquid handling software imports to read the values of the variables related to pipetting

\Logfile1.csv	‘this file stores data acquired over the time course of the experiment, including date/time, concentration of folAmix, optical density, growth rates,
