# Supplementary material for: Adaptation to mutational inactivation of an essential gene converges to an accessible suboptimal fitness peak
Source: eLife. 2019 Oct 1;8:e50509. doi: 10.7554/eLife.50509 (PMC6828540; doi:10.7554/eLife.50509)
Supplement: Transparent reporting form [file elife-50509-transrepform.docx]

***eLife’s* transparent reporting form**

We encourage authors to provide detailed information *within their submission* to facilitate the interpretation and replication of experiments. Authors can upload supporting documentation to indicate the use of appropriate reporting guidelines for health-related research (see [EQUATOR Network](http://www.equator-network.org/%20)), life science research (see the [BioSharing Information Resource](https://biosharing.org/)), or the [ARRIVE guidelines](http://www.plosbiology.org/article/info:doi/10.1371/journal.pbio.1000412) for reporting work involving animal research. Where applicable, authors should refer to any relevant reporting standards documents in this form.

If you have any questions, please consult our Journal Policies and/or contact us: [editorial@elifesciences.org](mailto:editorial@elifesciences.org).

**Sample-size estimation**

- You should state whether an appropriate sample size was computed when the study was being designed
- You should state the statistical method of sample size computation and any required assumptions
- If no explicit power analysis was used, you should describe how you decided what sample (replicate) size (number) to use

Please outline where this information can be found within the submission (e.g., sections or figure legends), or explain why this information doesn’t apply to your submission:

Since no clinical subjects are involved sample size issues in trial statistics are N/A.

Sample sizes for OD measurements of growth ranged from 10^4^ to 10^10^ bacterial cells per well in accordance with established relationship between OD and cell numbers in the volume of a well in 96-well plates.

Statistical analysis of protein and metabolite groupings resulting in p-values reported in Supplementary file 2 and Figure 2-figure supplement 3 and 4 is based on sample sizes of metabolites and proteins in each group ranging from 2 to about 200 as outlined in Supplementary file 2 and Supplementary file 3. Groups with small sample sizes typically featured high p-values of variation and were reported as insignificant.

**Replicates**

- You should report how often each experiment was performed
- You should include a definition of biological versus technical replication
- The data obtained should be provided and sufficient information should be provided to indicate the number of independent biological and/or technical replicates
- If you encountered any outliers, you should describe how these were handled
- Criteria for exclusion/inclusion of data should be clearly stated
- High-throughput sequence data should be uploaded before submission, with a private link for reviewers provided (these are available from both GEO and ArrayExpress)

Please outline where this information can be found within the submission (e.g., sections or figure legends), or explain why this information doesn’t apply to your submission:

Evolutionary trajectories were replicated 6 times for each initial strain as explained in Fig.1.

Growth rates of naïve, evolved and reconstituted strains D27G, D27C presented in Fig.3B are measured over 4 independent technical repeats, the plot shows average +/- SD over N=4 measurements.

Each metabolomics and proteomics experiment was performed for at least 3 biological replicates for each trajectory as outlined in Methods section p.20. Biological replicates represent separate clones plated from liquid culture and picked at random.

Biophysical measurements for DHFR variants presented in Table 1 were repeated at least 3 times as outlined in the legend to Table 1 and Methods pp.17-18. Errors reported in Table 1 are SD over 3 measurements.

WGS were carried out for selected clones followed by Sanger sequencing of relevant genes (*folA, thyA, deoB, upp* etc) for colonies selected from all trajectories at relevant time points as indicated in legend to Fig.5

**Statistical reporting**

- Statistical analysis methods should be described and justified
- Raw data should be presented in figures whenever informative to do so (typically when N per group is less than 10)
- For each experiment, you should identify the statistical tests used, exact values of N, definitions of center, methods of multiple test correction, and dispersion and precision measures (e.g., mean, median, SD, SEM, confidence intervals; and, for the major substantive results, a measure of effect size (e.g., Pearson's r, Cohen's d)
- Report exact p-values wherever possible alongside the summary statistics and 95% confidence intervals. These should be reported for all key questions and not only when the p-value is less than 0.05.

Please outline where this information can be found within the submission (e.g., sections or figure legends), or explain why this information doesn’t apply to your submission:

Statistical analysis of variation of protein levels in the TMT LC/MS proteomics experiments presented in Supplementary file 2 and Figure 2-figure supplement 3 and 4 was carried out as outlined in our previous publication Ref.14. That analysis included p-values and 95 confidence intervals based on statistical models explained in detail in Ref 14.

Statistical analysis of metabolic changes is presented in Fig.2, Fig.6 Figure 2-figure supplement 4, and Fig. 7. Z-scores are defined as outlined in results (p.6). p-values of metabolic variation color-coded in Figure 2-figure supplement 4 are explained in legend to Figure 2-figure supplement 4with reference to published software used to generate them. Error bars for concentration of metabolites presented in Fig.7 represent mean +/- SD taken over 3 technical repeats.

Statistical analysis of high throughput changes in metabolomes and proteomes presented in Fig.7AB represents Pearson linear regressions with corresponding p-values evaluated based on the number of data points for each regression

(For large datasets, or papers with a very large number of statistical tests, you may upload a single table file with tests, Ns, etc., with reference to sections in the manuscript.)

**Group allocation**

- Indicate how samples were allocated into experimental groups (in the case of clinical studies, please specify allocation to treatment method); if randomization was used, please also state if restricted randomization was applied
- Indicate if masking was used during group allocation, data collection and/or data analysis

Please outline where this information can be found within the submission (e.g., sections or figure legends), or explain why this information doesn’t apply to your submission:

N/A

**Additional data files (“source data”)**

- We encourage you to upload relevant additional data files, such as numerical data that are represented as a graph in a figure, or as a summary table
- Where provided, these should be in the most useful format, and they can be uploaded as “Source data” files linked to a main figure or table
- Include model definition files including the full list of parameters used
- Include code used for data analysis (e.g., R, MatLab)
- Avoid stating that data files are “available upon request”

Please indicate the figures or tables for which source data files have been provided:

All source data for Figs 2-7 are provided as Supplementary file 1,2,3,7
